# Report of the Corpus Christi Meeting of Association of Texas Health Officers

**Published:** 1908-08

**Authors:** 


### TEXAS MEDICAL JOURNAL.

Established July, 1885.

F. E. DANIEL, M. D., - - - - Editor, Publisher and Proprietor.

PUBLISHED MONTHLY.—SUBSCRIPTION $1.00 A YEAR.

VOL. XXIV. AUSTIN, AUGUST, 1908. No. 2.

The publisher is not responsible for the views of contributors.

### Department of Public Health.

### Report of the Corpus Christi Meeting of Association of Texas Health Officers.

(Continued.)

Collingsworth county represented by Dr. R. B. Wright was next heard from as follows:

Collingsworth county is situated in a very healthy portion of the State, and natural sanitary conditions are excellent as a rule. We have no large towns in the county. Wellington, the county seat, a town of one thousand inhabitants, has no slaughter houses or dairies. The court house is kept in good sanitary condition, but the court house yard needs improvements by way of providing closets, urinals, etc. A large closet in the corner of this yard is used by the public, and I regard this as being very unsanitary, and may result in the production and spread of disease if left in the present condition. I have advised improvement in this instance, but so far no action has been taken by the county commissioners to remedy this condition. This, I believe, to be from neglect rather than from any opposition upon the part of the commissioners court, as I think they will favor any reasonable expenditure to provide for betterment of these premises when forcibly reminded by the State Health Officer that it is their duty to do so. A Board of Health has been appointed by the commissioners court, consisting of three physicians residing in the town of Wellington. and we expect to inaugurate a vigorous cleanup campaign at an early date.

No particular attention is being given to the school buildings.

PURE FOOD.

No attention is being paid to milk and food inspection.

QUARANTINE.

There are now 22 cases of smallpox in the county. All under quarantine, and are of mild types except in three cases, but no deaths have as yet occurred from this disease. There is no leprosy, scarlet fever, diphtheria, cerebro-spinal fever, epidemic dysentery, typhoid or trachoma. I have no record of the tubercular cases, but know there is very few people so afflicted in this county. I have read the proposed sanitary code prescribed by you for unincorporated towns, and think that the citizens will heartily endorse the measures necessary when properly brought before them, which I, as County Health Officer, promise to do immediately after my return from this convention.

VITAL STATISTICS.

Practically all births are being reported, but think there is some negligence in reporting the deaths, but am unable to give per cent of unreported cases. I will add to this report that while in my section of the State we have had very little need for the enforcement of the sanitary laws, that our people endorse all measures calculated to be conducive to the public health, and commend the efficient services rendered this State by our present State Health Officer in localities where contagious diseases thrive.

Trinity county, represented by Dr. Jas. A. Hill, was next heard from, as follows:

It occurs to me disinfection is very important, and should be more thoroughly discussed by the county health officers before finally dismissing the patient; it occurs to me that next to prevention prior to communication is the proper disinfection immediately thereafter.

In the way of disinfection for this and similar occasions, I wish to call the attention of the county health officers to a very efficient and simple plan, and ask that you give this some thought and study, and if it is all that is claimed, we have certainly got an improvement over the older remedies in the way of convenience and simplicity than any others that I know, which means much to the busy man.

The preparation I will call your attention to is the permanganate disinfector, put up by Parke, Davis & Co., which to me is the ideal preparation, and by experimental research this method has proven to be very efficient and satisfactory.

This method is only a safe and more simple plan of liberating formaldehyde gas, and consists of a bottle of an official aques solution of formaldehyde (containing 40 per cent of absolute formaldehyde gas) and a box containing three briquettes, each briquette containing 80 grammes of potassium permanganate together with some kind of material to form a solid mass, and at the same time will permit reaction with the liquid to take place gradually.

This is put up in a neat box, and can be thrown in a buggy and carried along on the last visit, and after following the usual methods of cleaning up patient by bathing, etc., all you have to do is to simply close the room tight, pour the liquid into an ordinary galvanized bucket and drop the briquettes into liquid, withdraw from the room immediately, closing the door in the usual way to prevent the escape of gas. Leave the room unopened for twelve hours.

One package contains sufficient material for the disinfection of one thousand cubic feet under ordinary atmospheric conditions.

Personally, I believe permanganate disinfectant is an effectual method, and its simplicity makes it very convenient.

Dimmitt county, represented by Dr. R. B. Wright, was next heard from as follows:

Not much interest taken in sanitary affairs, as the locality is so far from railroad and we have had no practical education along these lines.

No organized forces, Carrizo Springs being the only town, and it having a small population and unincorporated.

No sanitary force except county health officer.

PURE FOOD.

No dairies in the county; butcher shops and hotels screened.

No deaths or cases during 1908 of the diseases mentioned.

Regulations would be complied with if the occasion should arise, as the county commissioners have left matter to the judgment of the county health officer.

No compulsory vaccination rules.

Have had no tuberculosis this year; one death in 1907, a transient.

I have read proposed code, but doubt being able to interest the citizens in any sanitary affairs except during the excitement of the actual presence of some contageous disease, at which time they are very enthusiastic in their advice to the authorities.

VITAL STATISTICS.

Approximately all cases of deaths and births are reported, those not reported being cases waited on by ignorant Mexican midwives. All the physicians obey the law strictly in making their reports.

In addition to the above, will state that the regulation regarding cleaning and sweeping of public buildings has been practically ignored by the county officials, school managers and church houses, although their attention has been called to the same by the county health officer. This rule has not been enforced by the county health officer on account of there not being any contagious disease in the county and the county being sparsely settled.

In regard to the disposal of fecal matter from public buildings without sewerage, I think it advisable to do away with underground vaults and make it compulsory on the county authorities to have receptacles for same, which shall be emptied and cleansed every few days, especially during the hot months. We have a vault in our county court house yard and it is a nuisance in every sense of the word.

Galveston county, represented by Dr. C. W. Trueheart, was next heard from as follows:

Lively interest in sanitary matters is manifested by the people of Galveston, as evidenced by the passage and rigid enforcement or ordinances requiring the screening of all cisterns with 18-mesh wire gauze, the treating with crude carbolic acid of all containers of water held for fire extinguishing purposes, and all open cesspools existing in the unsewered districts, and keeping petroleum on all undrainable standing water. By these means effecting and maintaining the extermination of mosquitoes, but especially the stegomyia fasciata mosquito, and thus divesting the once dreaded yellow fever of its terrors and the danger of its spread in the community. Ordinances requiring the systematic inspection of meat, fish, milk, fruits, vegetables, etc., and prohibiting the sale of meats, unless slaughtered in the up-to-date abattoir or like sanitary slaughter house. Prohibiting the sale of milk unless the milkman carries on both sides of his milk wagon a health office endorsement permit, a permit granted only to those whose dairies, cows and milk come up to the standard. Specimens of milk are systematically taken up by an intelligent milk inspector and regularly subjected to chemical analysis and microscopic examination by the city's bacteriologist.

The prompt report, under heavy penalty, by the attending physician and householders of all cases of yellow fever, smallpox, tuberculosis, bubonic plague, asiatic cholera, beri beri, typhoid fever, scarlet fever and diphtheria. Fumigation of the premises on removal or termination of cases of said disease, the burning of all bedding, etc., that can not be boiled, in cases of smallpox or bubonic plague.

I hold that in Galveston, or any other seaport where the extermination of the yellow fever spreading mosquito has been accomplished and is being maintained, that those features of quarantine most restrictive of commerce should be omitted or materially mitigated. This, I am confident, could be done without danger of communicating disease to the people of the port or to interior localities, even though the latter should be lax in the extermination of the mosquito.

### COST OF CARRYING ON GALVESTON CITY HEALTH DEPARTMENT FOR ONE YEAR.

| | |
|---|---|
| Salary Health Physician, $125, and horse feed, $10 per month, per year | $ 1,620 00 |
| Inspector Dairies and Food Stuffs, $85 per month, per year | 1,020 00 |
| Chief Health Inspector, $70, and horse feed $10 per month, per year | 960 00 |
| Scavenger Inspector, $70, and horse feed $10 per month, per year | 960 00 |
| Superintendent of Garbage Carts, $70, and horse feed $10 per month, per year | 960 00 |
| Two Routine Health Inspectors, $65, and horse feed $10 per month, per year | 1,800 00 |
| Two Routine Health Inspectors, 9 months at $65, and horse feed $10 per month, per year | 1,350 00 |
| Three distributors carbolic acid, 9 months at $65, and horse feed $10 per month, per year | 2,025 00 |
| One fumigator and keeper disinfectant store room, $50 | 450 00 |
| 14 garbage carts, average 26 days per month, 6 winter months at $3 per day | 6,552 00 |
| 18 garbage carts, average 26 days per month, 6 summer months at $3 per day | 8,424 00 |
| 2 dump tenders, $10 each | 240 00 |
| Budget allowance for carbolic allowance | 1,279 00 |
| Budget allowance for installation of garbage furnace | 2,500 00 |

| | |
|---|---|
| Incidental expenses, burying paupers, etc............ | 1,000 00 |
| Cost for caring of smallpox cases, yellow fever, etc. (comes out of special revenue fund) for 1907 was... | 2,455 31 |
| Garbage plant, recently appropriated............... | 15,000 00 |
| Total expenditures for year..................... | $ 47,595 31 |

Dr. Truheart continued as follows:

### THE MILK PROBLEM.

*Its Vital Importance and How Best to Solve It.*

No sanitary question of our day is fraught with graver importance to the health, happiness and well-being of all classes and conditions than the milk question.

*Milk an Active Absorbant.*

The milk and milk products are very liable to become impregnated with the taste and odor of stuffs with which they are brought in contact or even near, and that certain foods, among others, an excess of cotton seed meal, marsh grass, bitter weeds, etc., impart an unpalatable flavor to and impair the quality of milk is well known to dairymen, housewives and other handlers of milk.

*The Dangers from Infected Milk.*

That milk, when pure, is one of the best articles of food, especially for children, is an established fact, but that it is an active agent in absorbing and becoming contaminated with and conveying the germs of Asiatic cholera, diphtheria, measles, scarlet fever, tuberculosis, typhoid fever, venereal and other contagious diseases, has been well demonstrated and is recognized by up-to-date, scientific sanitarians the civilized world over.

*Rigid Inspection and Official Control the Only Corrective.*

Hence it stands to reason that the systematic inspection, official regulation and control, by milk inspectors and other public health officials, of the production and handling of this universally and largely consumed article of human food, from the time it leaves the healthy udder of the healthy cow until served upon the table of the consumer constitutes, by large odds, one of the most vitally important measures for preventing the spread of disease and promoting and safeguarding the public health.

### *Pasteurization Only a Partial Remedy.*

Pasteurization is advisable and efficacious only when the milk is consumed shortly after the process. Pasteurization of milk in bulk preliminary to distribution has proved disappointing in practical results secured.

### *Cleanliness the Absolute Necessity.*

Let it be steadily borne in mind that pasteurization or like process can not be substituted for cleanliness. That pure, wholesome milk can not be gotten out of the dairy when the cows are diseased or furnished with bad, unwholesome food or water; where the dairy and dairy equipment are not kept in a strictly sanitary condition; where the milkers and methods of handling the milk are not clean.

"Who can bring a clean thing out of an unclean? Not one." In this abhorism, that old-time philosopher, Job, uttered the keynote to modern sanitary science.

### *A Campaign of Education.*

In securing the effectual adoption of improved and perfected methods by the average dairyman, dairy employes and milk dealers, deeply-imbedded ignorance, prejudices and the force of long-practiced unsanitary methods and slovenly habits among these people have to be overcome.

A veritable campaign of education has to be waged; a school of systematic instruction has to be carried on by the milk inspector, and delinquents fined for violation of the laws.

### *Rules and Regulations.*

The fault—and it is a serious defect—with most of the sets of "rules and regulations" that have thus far been published for the instruction and guidance of people in the milk business, is that they deal too exclusively in generalities—don't go sufficiently into the smaller details, which really constitute the gist, the important whole to be inculcated; fail to specify just what constitutes good sanitation; leave too many points to the crude, untutored judgment of ignorant people.

### *The Galveston Plan.*

Galveston Health Department furnishes to each and all dairymen and handlers of milk a set of "rules and regulations" printed in large type on durable cloth. These placards are framed and conspicuously posted in every dairy and dealer's premises. In these "rules and regulations" every step, even to the smallest detail, is described and ordered. The city ordinance makes each employe, as well as the owner, responsible and punishable for the violation of any of these rules.

This provision has been found to exert a most beneficial effect.

Specimens of the placard, etc., will be cheerfully furnished health officers applying for them.

Harrison county, represented by Dr. J. Holman Taylor, was next heard from, as follows:

The city of Marshall is incorporated, has its own sanitary laws and officers, and is excluded from the scope of this report.

The towns of Hallsville, Waskom, Jonesville, Scottsville and Woodlawn, are not incorporated, and have no sanitary code or special officers. They are all thriving, wide-awake communities, and might be induced, after proper explanation, to incorporate for sanitary purposes. They are all uniformly healthy communities, though, and that fact would militate against the agreement referred to.

I believe the people of this county are above the average in appreciation of proper efforts at sanitation. Certain it is that the commissioners court is alive to the good to be derived thereby. Our jail and court house are in good sanitary condition, and some money has been expended in making them so.

There is no attempt on the part of the county to inspect either dairy or food products, but I feel sure they would co-operate with towns or cities in any reasonable effort to control these matters.

There have been reported and quarantined one hundred cases of smallpox since January 1st, many of which, as a matter of course, occurred in quarantine. Only one death has resulted from all of these cases and that one was complicated with a recent childbirth. The type of the disease has varied from an exceedingly mild discreet variety to the confluent form. No other diseases have been reported to this office.

The modified form of quarantine has been adopted, and has proven very satisfactory. The commissioners court has complied with all requirements to insure a rigid quarantine, and has authorized me to use whatever means I think best in securing same. As a rule, the regulations are adhered to by those affected; in a few instances only have they been violated, and in these cases speedy conviction and punishment was secured. Vaccination is not a requirement for entrance into our public schools, but we secure vaccination when it is specially desirable by threatening to quarantine suspects or by closing schools.

No special attention is paid tuberculosis by the health department of this county. It is not reported to us at all.

Our people are especially well informed as to the damage done by the mosquito, and its extermination is attempted wherever there seems to be a fair chance of accomplishing anything. Under the conditions you name I would not advocate a hard and fast quarantine, and I feel sure our people here would abide by your advice in the premises when backed by mine. But assurance would have to be doubly sure to overcome the dread of that disease inspired by the disastrous epidemic of 1873, which decimated this place.

The City of Marshall, represented by Dr. R. C. Hall, was next heard from, as follows:

Our people are very much interested in sanitary affairs, and the city is as clean as coud be reasonably desired. Our market houses are all screened and in good sanitary condition. All slaughter houses are outside of the city limits, and generally removed from closely populated neighborhoods. No attention is being paid to the milk supply. The sanitary force of the city consists of health officer, sanitary officer, ten men and three teams, together with city prisoners when needed, all constantly occupied except health officer.

No effort is being made in the line of milk or food inspection.

There have been twenty-two cases of smallpox in the city since January 1st, with no deaths. One case of diphtheria, no death. No leprosy, scarlet fever, cerebro-spinal meningitis or trachoma. Quarantine regulations, isolation and disinfection are complied with in all cases of smallpox, scarlet fever and diphtheria. In disinfecting two pounds of sulphur per 1000 cubic feet are used, and house kept sealed for four to six hours. Physicians are cooperating with us in this matter, and the council has authorized me to use all reasonable means of protecting the community from spread of the diseases mentioned. Vaccination is not compulsory as a matter of entrance requirements to our public schools.

Physicians are not reporting tuberculosis, and if infected houses are being fumigated it is not being done by the city. I am of the opinion, however, that a large per cent of infected houses are disinfected after the death of patient, as the physicians of the community are rather active in the dissemination of knowledge regarding the methods of spread of tuberculosis, and the people know enough of the ravages of the disease to wish to stop it. So far as I am able to judge, there has been no perceptible increase in percentage of cases of tuberculosis in the city in the past five years.

Our people are alive to the necessity for mosquito extermination, and are using oil on all stagnant water, screening all cisterns and houses and draining flats as well as possible. I think a yellow fever patient brought to a place well screened, and kept so, is perfectly harmlesss, but I am not so sure that our people would be content to accept my word for it, though they and our officials seem to have every confidence in my judgment. The great loss of life from this scourge in this place in 1873 developed the shotgun quarantine as a protective measure, and its efficiency is so well known that I doubt whether they would be willing to give it up or not, even upon the assurance of our most scientific sanitarians.

I am of the opinion that most of our physicians report all vital statistics, and I would judge that midwives report practically all of their births.

Liberty county, represented by Dr. S. M. Jordan, was next heard from as follows:

We have no regular organized sanitary force in our county, and only one incorporated town. The people generally realize the importance of screening and, therefore, there has been a recent addition made to our jail with modern appliances and the court house is kept cleaned up in first-class condition and a disinfectant fluid is used in the spittoons, and the floors are scrubbed once or twice a week. Our schools in the county are being kept in fairly good sanitary order; there is no compulsory vaccination for children entering school. So far we have not had any smallpox in that section since January; as to diphtheria, scarlet fever, trachoma epidemics and cerebro-spinal meningitis, they have not been known; as to leprosy, we have one old case of it about twelve to fifteen years' standing among the French located about five miles from the city of Liberty. This case has been previously reported to Dr. Tabor, and, no doubt, had its origin in Louisiana. So far as I can say, don't think there is any new cases; but under the conditions that these people are living, their associations are such that it is not at all improbable that there will be some new cases develop within the near future. This patient is a pauper and is taken care of by the county health officer. I have never tried to administer as to the cure of the disease, only prescribing emollient salves. I have not heard of any typhoid since the first of January, but some para-typhoid cases. I have read the sanitary code, and I agree with you that it would be unnecessary to put on strict quarantine in case yellow fever should break out where the proper precautions are taken at the points of infection to prevent its spread. The commissioners court has not yet given me permission to take full control of cases without their order, but have promised that they would abide by the rules promulgated and endorsed by the conference now in session. As to reporting of births and deaths, there are about thirty doctors in the county with not more than ten who make prompt reports. There have been five deaths from tuberculosis in the county since the first of January, and at presen I know of no new cases in the county; but urge physicians on every occasion to make the report of all infectious diseases. The houses are disinfected thoroughly by the physicians in charge. I do not know the exact number of deaths and births since January 1st, as there have been quite a few to my knowledge that have not been reported. There is no effort being made to inspect milk and food. The milk supply comes from families who own their cows, and in case they get more than they use they sometimes sell to their neighbors, and I don't think that it is necessary for such inspection to be carried on in the county. Realizing that the compensation to health officers is inadequate for the services rendered, would be glad that this conference pass some resolution so as to enable us and our successors to be more amply compensated for services rendered in the future.

